# Draft Genome Sequence of Lactobacillus jensenii UMB0847, Isolated from the Female Urinary Tract

**DOI:** 10.1128/MRA.00395-20

**Published:** 2020-05-14

**Authors:** Ella West-Pelak, Taylor Miller-Ensminger, Adelina Voukadinova, Alan J. Wolfe, Catherine Putonti

**Affiliations:** a Neuroscience Program, Loyola University Chicago, Chicago, Illinois, USA; b Bioinformatics Program, Loyola University Chicago, Chicago, Illinois, USA; c Department of Microbiology and Immunology, Stritch School of Medicine, Loyola University Chicago, Maywood, Illinois, USA; d Department of Biology, Loyola University Chicago, Chicago, Illinois, USA; e Department of Computer Science, Loyola University Chicago, Chicago, Illinois, USA; University of Southern California

## Abstract

Lactobacillus jensenii is a protective bacterium native to the female urinary tract. Here, we present the 1.6-Mbp draft genome for Lactobacillus jensenii UMB0847, isolated from a catheterized urine sample obtained from a pregnant female.

## ANNOUNCEMENT

Lactobacillus jensenii, along with L. crispatus, L. gasseri, and L. iners, is frequently found within the microbiota of the female urogenital tract (1, 2). It is believed to be involved in maintaining a healthy urogenital tract (1–4). Many strains of L. jensenii produce hydrogen peroxide, which can be toxic to other organisms (5). Previous studies have shown that many lactobacilli, including L. jensenii, are effective in displacing or inhibiting the growth of urogenital pathogens, including Escherichia coli (6, 7), Enterococcus faecalis (8), and Neisseria gonorrhoeae (9). Analysis of L. jensenii genomes from the vaginal microbiota has yielded evidence of adaptation to this particular environment (3). Recently, we began sequencing L. jensenii representatives of the urinary microbiome (10). Here, we introduce the draft genome of another L. jensenii strain from the urinary tract, L. jensenii UMB0847, which was isolated from a pregnant female.

L. jensenii UMB0847 was isolated from a prior institutional review board (IRB)-approved study (11) using the expanded quantitative urinary culture (EQUC) protocol (12). The genus and species were confirmed by matrix-assisted laser desorption ionization–time of flight (MALDI-TOF) mass spectrometry, as previously described (12), and then stored at −80°C until sequencing. We streaked the L. jensenii isolate onto a Columbia nalidixic acid (CNA) agar plate and incubated it at 35°C in 5% CO_2_ for 24 h. A colony was selected to grow in liquid brain heart infusion (BHI) medium at 35°C in 5% CO_2_ for 24 h. DNA was extracted using the Qiagen DNeasy blood and tissue kit following the manufacturer’s protocol for Gram-positive bacteria with the following exceptions: we used 230 μl of lysis buffer (180 μl of 20 mM Tris-Cl, 2 mM sodium EDTA, and 1.2% Triton X-100 and 50 μl of lysozyme) in step 2 and altered the incubation time in step 5 to 10 min. The extracted DNA was quantified using a Qubit fluorometer and sent to the Microbial Genomic Sequencing Center at the University of Pittsburgh for sequencing. DNA libraries were constructed using the Nextera XT kit. The DNA was sequenced using the Illumina NextSeq 550 platform, producing 3,260,770 pairs of 150-bp reads. These raw reads were trimmed using Sickle v1.33 (https://github.com/najoshi/sickle). We used SPAdes v3.13.0 (13) with the “only-assembler” option for k values of 55, 77, 00, and 127. We used PATRIC v3.6.3 (14) and the NCBI Prokaryotic Genome Annotation Pipeline (PGAP) v4.11 (15) to annotate the genome. Genome coverage was calculated using BBMap v38.47 (https://sourceforge.net/projects/bbmap/). Unless specifically noted, all software tools used default parameters.

The L. jensenii draft genome has a length of 1,644,700 bp and consists of 44 contigs with a GC content of 34%, genome coverage of 485×, and an *N*_50_ score of 57,991 bp. The PATRIC annotation identified 1,044 genes that produce functional proteins, 54 tRNAs, and 6 rRNAs (3 5S, 2 16S, and 1 23S). Furthermore, 1 CRISPR array containing 12 spacer sequences was detected. Continued investigation of L. jensenii from the urinary microbiota will increase our knowledge of the genetic diversity of this beneficial member of the female urogenital tract microbiota.

### Data availability.

This whole-genome shotgun project has been deposited in GenBank under accession no. JAAUWK000000000. The version described in this paper is the first version, JAAUWK010000000. The raw sequencing reads have been deposited in the SRA under accession no. SRR11441018.
